# Paramedic and midwifery student exposure to workplace violence during clinical placements in Australia – A pilot study

**DOI:** 10.5116/ijme.582e.ac04

**Published:** 2016-12-11

**Authors:** Malcolm Boyle, Lisa McKenna

**Affiliations:** 1 School of Medicine, Griffith University, Queensland, Australia; 2 School of Nursing and Midwifery, Monash University, Victoria, Australia

**Keywords:** Paramedic, midwife, nurse, student, workplace violence

## Abstract

**Objectives:**

The objective of this pilot study was to identify the type of workplace violence experienced by undergraduate paramedic and midwifery students.

**Methods:**

The study used a cross-sectional methodology with the self-administered paper-based Paramedic Workplace Violence Exposure Questionnaire to elicit undergraduate paramedic and midwife responses to workplace violence whilst on clinical placements. There were 393 students eligible for inclusion in the study. A convenience sample was used. The anonymous questionnaire took 10 to 20 minutes to complete. Descriptive statistics are used to summarise the data with a two-tailed t-test used to compare groups.

**Results:**

The main form of workplace violence was verbal abuse 18% and intimidation 17%. There was a statistically significant difference between midwifery and paramedic students for intimidation (t_(134)_=-3.143, CI: -0.367 to -0.082, p=0.002) and between females and males for sexual harassment (t_(134)_=2.029, CI: 0.001 to 0.074, p=0.045), all other results were not statistically different.

**Conclusions:**

This pilot study is the first of its kind in Australia and internationally to identify exposure rates of workplace violence by undergraduate paramedic students during clinical placements and one of very few to identify midwifery students’ exposure rates of workplace violence. The study identified that students were exposed to a range of workplace violence acts from verbal abuse through to sexual harassment. These findings highlight a need for investigation of workplace violence exposure of medical, nursing and allied health students during the clinical phase of their studies.

## Introduction

Studies conducted in Australia, the United Kingdom, and the United States have reported occupations with significant face-to-face contact were particularly at risk of patient initiated violence.^1^  A report for the International Labour Organisation found females experienced higher levels of verbal and sexual abuse whereas males experienced more overt threats and physical assaults.^2^

From our experience, acts of workplace violence against students have been verbally reported to faculty. However, students generally have not wished to continue with the reporting process when asked.  Anecdotally, we know that paramedic and midwifery students will not report acts of workplace violence during clinical placements as they do not want to jeopardise their opportunity of getting a job with the respective employer.  In turn, the ambulance service and hospitals will not act upon reported workplace violence that occurred during the clinical placement unless it is in writing.

Internationally, no study has been published that has investigated the exposure rate of undergraduate paramedic students to acts of workplace violence, whilst there has been two studies reporting midwifery student exposure to workplace violence.^3^^,^^4^  A Turkish study by Lash and colleagues investigated midwifery student exposure to various types of verbal abuse and their subsequent response, but due to the study type, a phenomenological study, no exposure rates were reported. Of the midwifery students eligible to participate in the study 90% elected to do so.  Data were collected during focus group sessions with the questions seeking information about experience and response to verbal abuse.  There were six themes about verbal abuse which were identified from the data with students acknowledging that being young, university educated, lacking experience, and a midwife/nurse as some of the precipitating factors leading to verbal abuse against them.^4^

The second study into bullying of midwifery students was undertaken by Gillen and colleagues in the United Kingdom (UK), where they examined the exposure of midwifery students to workplace bullying.  The study by Gillen and colleagues surveyed midwifery students at a conference and obtained a 41% return rate.  The majority of students were over 30 years of age with approximately two-thirds of them married or living with their partner.  Just over a third of students reported being bullied and another third reporting they had witnessed another person being bullied.  The perpetrator in at least half of the episodes was a midwife or mentor.^3^

This research sought to identify the magnitude of workplace violence to undergraduate paramedic and midwifery students during clinical placements. Therefore, the objective of this pilot study was to identify the type of workplace violence experienced by undergraduate paramedic and midwifery students. 

## Methods

### Study design

This pilot study used a cross-sectional methodology (survey) in the form of a self-administered paper-based questionnaire to elicit paramedic and midwifery students’ responses to acts of workplace violence against them whilst on clinical placements.

### Definitions

We utilised the International Labour Office (ILO) definition for violence, where violence is defined as:

“The intentional use of physical force or power, threatened or actual, against oneself, another person, or against a group or community, which either results in or has a high likelihood of resulting in injury, death, physiological harm, maldevelopment, or deprivation.”^5^

In addition, we also employed the definition for workplace violence published by the World Health Organisation (WHO) and ILO in the Framework Guidelines for addressing workplace violence in the health sector:

“Workplace violence covers a spectrum of unacceptable behaviours. It includes incidents where staffs are abused, threatened, discriminated against or assaulted in circumstances related to their work, including commuting to and from work, and which represent a threat to their safety, health, and well-being.”^6^

For this study the workplace included the ambulance station or hospital, offices where management were housed, the ambulance itself, any ward or room associated with the hospital, any other healthcare facility, and for the paramedic students the incident/case location. Workplace violence acts are defined in Appendix 1.

### Participants

Study participants were drawn from students enrolled in the Bachelor of Emergency Health (Paramedic) [BEH], Bachelor of Nursing/Bachelor of Emergency Health (Paramedic) [BN/BEH], and Bachelor of Midwifery (BMid) courses at Monash University, Peninsula Campus.  The Bachelor of Emergency Health (Paramedic) is an undergraduate course for paramedics working in the ambulance service in Victoria, Australia.

There were a total of 393 students eligible for inclusion into the study with 132 BEH students, 158 BN/BEH students and 103 BMid students.  Second year BEH students were not included as they did not undertake any clinical placements during the first semester. Students in the third year of the BN/BEH course undertake the same clinical placements as first year BEH students with fourth year BN/BEH students undertaking the same clinical placements as third year BEH students.

A convenience sample of students was used to establish an incident rate for further study. The overall response rate was 34.6% (136 out of 393) with the response rate for BEH, BN/BEH and BMid students being 60 (45%), 24 (15%) and 52(50%) respectively. Overall there were 29 (21.3%) males and 107 (78.7%) females.  There were 24 (40%) males and 36 (60%) females in the BEH group, five (20.8%) males and 19 (79.2%) females in the BN/BEH group, and 52 (100%) females in the BMid group.

Ethics approval for the study was granted by the Monash University Human Research Ethics Committee (MUHREC).

### Instrument

We utilised an existing paper-based self-administered questionnaire, the Paramedic Workplace Violence Exposure Questionnaire (PWVEQ).^7^ The PWVEQ consists of five sections. The first section covered general demographic information, including the course being studied, year of the course, age, and gender.  The second section covered five forms of workplace violence - verbal abuse, intimidation, physical abuse, sexual harassment, and sexual assault.  The information sought for each act of violence included exposure to the violent act, the frequency of exposure, the location of the act, e.g. work location, private residence, public place, the perpetrator of the act, the perpetrator’s gender, potential underlying factors, e.g. alcohol intoxication, and the immediate response and the level of fear.  The third section covered how the student felt personally after experiencing an episode of violence against them.  The information sought was free text about the impact on the person themselves, personal relationships, and their work.  The fourth section covered the response to the violent incident(s).  The information sought was in a Likert Scale, ranging from “never” to “always”, about how the student responded to the violence act.  The final section covered the impact of the violence.  This utilised the Impact of Event Scale, a Likert Scale ranging from “not at all” to “often”.^8^

The PWVEQ has been used previously in a study with paramedics and demonstrated face and content validity.^7^   This questionnaire was chosen for use with midwifery students, as the questions were sufficiently generic for both student groups, as it contained no discipline specific questions.

### Procedures

Students were informed about the study at the end of a lecture by a non-academic staff member and were invited to stay and participate in the study.  All participating students received an explanatory statement about the study and were informed participation was voluntary and anonymous prior to commencing the survey. Consent was implied by completion and submission of the anonymous survey into a drop box.  Students were advised they could withdraw from the study prior to submitting the questionnaire, due to the anonymous nature of the questionnaire they could not withdraw once the questionnaire had been placed in the drop box.  The questionnaire took 10-20 minutes to complete; this was dependent upon their exposure to acts of workplace violence.

Data were extracted from the completed survey forms and entered into an Excel™ spreadsheet by a research assistant, the data entry was cross checked by one of the authors (MB) prior to uploading into the statistical program.

### Data analysis

Data analysis was undertaken using SPSS.  Descriptive statistics were used to summarise the data, student course, year of the course, student age, student gender, rate of abuse, and frequency of abuse.  Inferential statistics were used to compare groups (violence act type, paramedic or midwife student, and gender) using a t-test.  All results are two-tailed unless otherwise stated. The results will be considered significant if the p value is <0.05, all confidence intervals (CI) are 95%. 

## Results

The average age of all students was 26 years, median age of 23 years.  For the BEH group mean age was 24.7 years, median age 23 years, BN/BEH group, mean age was 23.5 years, median age 22 years, and BMid group mean age was 28.7 years, median age 25.5 years. The majority of students were in third year with the fourth year students from the BN/BEH course, see Table 1.

**Table 1:** Study participants by course by year

Course	Yr1 n (%)	Yr2 n (%)	Yr3 n (%)	Yr 4 n (%)	Total n
Emergency Health (Paramedic)	21 (35)	N/A	39 (65)	N/A	60
Nursing/ Emergency Health (Paramedic)	0	0	0	24 (100)	24
Midwifery	21 (15)	21 (15)	69 (52)	N/A	52

Table 2 demonstrates that the most common form of violence experienced by students overall was verbal abuse followed by intimidation.  There were a small number of sexual harassment incidents, and no incidence of sexual assault.  The only statistically significant result was the comparison between paramedic and midwifery students who experienced intimidation, all other comparisons between paramedic and midwifery students were not statistically significant.  These results indicate that midwifery students experienced more acts of violence against them during their clinical placement compared to the paramedic students.

**Table 2:** Number of students who experienced each type of violence during their last clinical placement

Violence Type	Overall	Paramedic Students (n=84), n (%)	Midwifery Students (n=52), n (%)	Difference between Paramedic and Midwifery Students
Verbal abuse	24	16 (66.7)	8 (83.3)	t_(133)_=0.572, CI: -0.096 to 0.174, p=0.568
Intimidation	23	7 (30.4)	16 (69.6)	t_(134)_=-3.143, CI: -0.367 to -0.082, p=0.002
Physical Abuse	1	1 (100)	0 (0)	t_(131)_=0.763, CI: -0.019 to 0.043, p=0.447
Sexual Harassment	4	1 (25)	3 (75)	t_(134)_=-1.318, CI: -0.115 to 0.024, p=0.192
Sexual Assault	0	0 (0)	0 (0)	

**Table 3:** Students experiencing violence type by gender

Violence Type	Female (n=107)	Male (n=29)	Difference between female and male
Verbal Abuse	18 (75%)	6 (25%)	t_(133)_=-0.46, CI: -0.197 to 0.122, p=0.646
Intimidation	19 (82.6%)	4 (17.4%)	t_(134)_=0.502, CI: -0.117 to 0.196, p=0.617
Physical Abuse	1 (100%)	0 (0%)	t_(131)_=0.527, CI: -0.027 to 0.046, p=0.599
Sexual Harassment	4 (100%)	0 (0%)	t_(134)_=2.029, CI: 0.001 to 0.074, p=0.045
Sexual Assault	0 (0%)	0 (0%)	

Of the students surveyed 32% had experienced at least one form of violence associated with the clinical placement, with 81% of these students being female. The results in Table 3 demonstrate that more female than male students experienced sexual harassment.  All other comparisons between male and female students were not statistically significant.

Students were asked to indicate the frequency of workplace violence during their last set of clinical placements. The results are presented in Table 4.  It is clear that students experienced verbal abuse and intimidation on more than one occasion.

**Table 4:** Frequency with which each type of abuse was experienced

Violence Type	Once n (%)	A few times n (%)	About once a month n (%)	About once a week n (%)	Daily n (%)
Verbal Abuse	10 (7.4)	10 (7.4)	3 (2.2)	2 (1.5)	-
Intimidation	11 (8.1)	9 (6.6)	2 (1.5)	1 (0.7)	-
Physical Abuse	1 (0.7)	-	-	-	-
Sexual Harassment	-	4 (2.9)	-	-	-
Sexual Assault	-	-	-	-	-

## Discussion

This pilot study was the first of its kind in Australia and internationally to investigate the exposure to workplace violence by undergraduate paramedic students during clinical placements and one of very few to investigate midwifery students’ exposure to workplace violence.  This pilot study has revealed undergraduate paramedic and midwifery students are exposed to various types of workplace violence during their clinical placements.

The reporting of workplace violence against paramedics dates back to the early 1990s where a study in the United States of America (USA) found paramedics were exposed to violence in the workplace.^9^ For nursing students, a study in the USA published in 1996 appears to be the first into workplace violence experienced by nursing students.^10^ The reporting of midwifery student exposure to workplace violence commenced in 2006 with the latest in 2016.^4^^, ^^11^

When reviewing the results of exposure to violence for paramedic students, the numbers appeared low in comparison to the results from a Victorian and South Australian study.^7^  However, when examining the time for possible exposure it was found that the students were exposed to less hours than a practising paramedic would be in a twelve month period.  First year students had a 1% and third years a 10% possible exposure time compared to that of a practising paramedic.  This explains the lower than expected workplace violence rate for paramedic students.  However, when the student clinical placement time is extrapolated out to the time that a paramedic would normally be working over a twelve month period the student exposure rate proved to be similar to that reported in the Victorian and South Australian study.^7^

The reporting of midwives exposure to workplace violence dates back to 1996, when a study commissioned by the Royal College of Midwives reported on the rate of bullying against midwifes.^12^ When examining the time for possible exposure, again, it was found that the midwifery students were exposed to less hours than a practising midwife would be in a twelve month period.  Second year students had a 12% and third years a 26% possible exposure time compared to that of a practising midwife. Similar to the paramedic students, when the midwifery student time is extrapolated out to the time that a midwife would be working over a twelve month period, the student exposure rates proved to be similar to that reported by Yashida and Sandall.^13^

As there is no international data on paramedic student exposure to workplace violence we will use the Australian studies as a comparator.  International data on workplace violence exposure of midwifery students is limited therefore we will use the studies identified plus studies reporting on nursing students’ exposure to workplace violence.

The incidence of verbal abuse against paramedics internationally has varied from 21% to 78%.^14^^-^^16^ In this study we found that 67% of paramedic students had been verbally abused. This verbal abuse rate against paramedic students appears high, given the short time they were exposed to the environment during clinical placements.  Verbal abuse is the most common form of workplace violence that may have a long lasting effect on the paramedic student.  Given the range of cultures that paramedic students belong to, racial abuse was not part of the questioning in the survey, which should be a consideration in further studies.  Racial verbal abuse is a general form of abuse that is not restricted to the working environment, which may affect different students in a variety of ways and may prove difficult to measure.

The incidence of verbal abuse against midwifery students internationally has been reported at 100% in one study 4, where in this study we found 83.3% of midwifery students had been verbally abused.  Verbal abuse against nursing students in various international locations varies from 17%11 to 100%17.  Verbal abuse towards midwifery and nursing students is primarily perpetrated by medical staff and nursing supervisors, especially in countries that do not have strict workplace laws. 

In this study, exposure to intimidation was higher in midwifery students compared to paramedic students, 69.6% to 30.4%. This higher percentage for midwifery students is also seen in other nursing related studies from New Zealand18 at 90%, and the United Kingdom3 at 63%.  It would appear the nursing and midwifery students are intimidated more by medical staff and nursing supervisors in a “power play” to demonstrate the control they have over the student as a result of their position. Intimidation may be a precursor to sexual harassment/assault however we were unable to determine this fact from the questionnaire data and would require a prospective study and some focus group work to help confirm this.

The incidence of physical violence in this study was low, none reported for midwifery students and only one incident for paramedic students, whereas international nursing student studies have reported physical violence ranging from 4.2%19 to 15.6%20.  We would have expected the paramedic students to be exposed to more physical violence because of the uncertain environment in which they operate.  The low physical violence rate in this study may be attributed to the shifts the students worked they only work day shifts, where most physical violence occurs during the night shifts, after 6pm.  The paramedic students may also be protected more by the paramedic crews they are working with, hence the low rate of physical abuse.

The rate of physical violence reported in international paramedic studies is predominately much higher, 2.9% to 79.5%. Several studies from the USA specifically reported physical assault with a weapon, we did not specifically ask about assault with a weapon.  Indeed, assault with weapons on paramedics appears to be a rare occurrence in Australia compared to other countries such as the USA.^9^^, ^^14^^-^^16^^, ^^21^^-^^23^

The incidence of sexual harassment was low, 7%, with no reports of sexual assault in this study. In this study, sexual harassment of paramedic students made up 4% and midwifery students 7% of the total violence exposure. In international studies, sexual harassment ranged from 4.2% to 57% for nursing and midwifery students.^11^^,^^17^^, ^^19^^, ^^20^^, ^^24^ In this study, students exposed to sexual harassment were all females with only one international study identifying that males were also exposed to sexual harassment.^20^

The implications of the findings from this study highlight a need for the education of undergraduate students in how to handle and cope with workplace violence. Currently there is no formalised education about WPV within either of the undergraduate paramedic or midwifery curricula at Monash University.  Paramedic students can undertake their first clinical placement within the first four to six weeks of year one of the course.  This clinical placement is not necessarily related to the subjects they are studying at the time but to show them the work of a paramedic.  This makes it difficult to include a specific lecture or information session on WPV and how to handle it, therefore a standalone, short information session needs to be developed alongside a reporting system. There is also a need to develop a reporting system that students are willing to use so that faculty are aware of the exposure to an act of workplace violence thereby ensuring follow up of the student. Overall, there needs to be better education of students about the acts of WPV, how to handle the acts of violence against them, and the importance of reporting exposure to acts of WPV.

This study is potentially limited by the ability of students to recall incidents of workplace violence.  Furthermore, using a single method of measurement (i.e. self-reports of a past period, up to several months ago) without corroboration from other external sources of information such as observer-ratings, may have limited the validity of our findings.  Moreover, the retrospective nature of the study and small sample size may mean the results are not necessarily a true representation of the total paramedic and midwifery student population at Monash University or within other Australian tertiary institutions.

## Conclusion

The results of this pilot study demonstrate that undergraduate paramedic and midwifery students are exposed to acts of violence when undertaking their clinical placement.  The study identified that students were exposed to a range of workplace violence acts from verbal abuse through to sexual harassment.  These results also highlight a need for student education prior to attending the clinical placement on how to deal and cope with workplace violence and the importance of reporting workplace violence exposure.  Further research is required in this area to better understand the precipitating factors of workplace violence against all students.

### Acknowledgements

This study was funded by the Monash University Peninsula Campus Formative Research Grant.We like to thank the students who took the time to complete and return the survey.

### Conflict of Interest

The authors declare that they have no conflict of interest.

## Supplementary material

Supplementary file 1 Appendix 1. Types of violence definitions
