# Supplementary material for: Paramedic and midwifery student exposure to workplace violence during clinical placements in Australia – A pilot study
Source: Int J Med Educ. 2016 Dec 11;7:393–9. doi: 10.5116/ijme.582e.ac04 (PMC5149425; doi:10.5116/ijme.582e.ac04)
Supplement: Supplementary file 1 — Appendix 1. Types of violence definitions [file ijme-7-393-S1.pdf]

**Appendix 1**

Types of violence definitions

| Violence Type | Definition |
|---|---|
| Verbal abuse | A patient/client, their friend/s, family member/s, other professional/s or work colleague/s using offensive language, yelling or screaming with the intent of offending or frightening you. It can include threats or abuse over the phone, but excludes sexual harassment and sexual assault. |
| Intimidation | A patient/client, their friend/s, family member/s, other professional/s or work colleague/s purposely threatening, following you, using gestures to purposely offend or frighten you. |
| Physical abuse | A patient/client, their friend/s, family member/s, other professional/s or work colleague/s physically attacking you, or attempting to attack you. It includes behaviours such as punching, slapping, kicking or using a weapon or other object with the intent of causing bodily harm. |
| Sexual harassment | Any form of sexual propositioning or unwelcome sexual attention from a patient/client, their friend/s, family member/s, and other professional/s or work colleague/s. It includes behaviours such as humiliating or offensive jokes and remarks with sexual overtones, suggestive looks or physical gestures, inappropriate gifts or requests for inappropriate physical examinations, pressure for dates, and brushing, touching or grabbing excluding sexual touching (e.g. the genital or breast area). |
| Sexual assault | Any forced sexual act, rape or indecent assault perpetrated by a patient/client, their friend/s, family member/s, and other professional/s or work colleague/s. It includes brushing, touching or grabbing of the genitals or breast. It also includes attempted sexual assault. |
